# “*I can be pro-abortion and pro-birth*”: Opportunities and challenges for full spectrum care among doulas in Georgia

**DOI:** 10.3389/fgwh.2023.966208

**Published:** 2023-03-01

**Authors:** Alyssa Lindsey, Subasri Narasimhan, Ayeesha Sayyad, Daria Turner, Elizabeth A. Mosley

**Affiliations:** ^1^ Rollins School of Public Health, Emory University, Atlanta, GA, United States; ^2^ The Center for Reproductive Health Research in the Southeast, Emory University, Atlanta, GA, United States; ^3^ Department of Health Policy and Behavioral Sciences, Georgia State University, Atlanta, GA, United States; ^4^ Division of General Internal Medicine, Center for Innovative Research on Gender Health Equity (CONVERGE), University of Pittsburgh School of Medicine, Pittsburgh, PA, United States

**Keywords:** abortion, contraception, doula, birth worker, stigma, family planning, full spectrum doula, abortion doula

## Abstract

**Background:**

The work of full spectrum doulas (i.e., non-medically trained care workers offering support before, during, and after pregnancy including abortion)—is increasingly important as abortion access decreases across the U.S. Few studies have examined the work of community-based doulas in restrictive abortion settings or how they might further incorporate full spectrum care. As part of the community-engaged mixed methods Georgia Doula Study, this analysis examines the scope of work of community-based doulas regarding full spectrum and abortion services, doula opinions on full spectrum and abortion work, and potential barriers and facilitators for full spectrum doula care in metro-Atlanta, Georgia.

**Methods:**

From October 2020 to February 2022, the team recruited 20 community-based doulas with 8 who provide full spectrum services including abortion. Surveys covered demographics, doula scope of work, family planning attitudes, and abortion stigma. Survey data were analyzed using descriptive and bivariate statistics. In-depth interviews further explored those topics. They were de-identified and thematically analyzed using a semi-deductive approach.

**Results:**

The findings are organized around five themes: (1) doulas of all kinds center reproductive autonomy; (2) abortion doulas play important roles in reproductive autonomy; (3) doulas have mixed feelings about contraceptive counseling; (4) abortion doulas provide diverse services carrying numerous benefits in a stigmatized environment; and (5) abortion doulas experience challenges including stigma but they offer solutions. All but two doulas in this study were interested in learning how to incorporate contraception and abortion services in their current scope of work, and most participants supported the role of full spectrum doulas.

**Conclusion:**

This analysis highlights the experiences of abortion and full spectrum doulas, reactions of the larger doula community to those services, and facilitators and barriers to full spectrum doula care in a restrictive abortion setting. There are urgent needs and opportunities for full spectrum doulas to offer life-protecting services to pregnant people across the U.S. and globally. Coordination efforts for U.S. abortion care post-*Roe v. Wade* must include community-based doulas, who are largely open to aiding abortion clients through education, connection to care, and emotional support.

## 1. Introduction

Following the Supreme Court of the United States' decision to overturn *Roe v. Wade* and federal protection of abortion rights (1), each U.S. state now has the authority to regulate and even outlaw abortion. In turn, restrictive and harmful abortion bans were rapidly implemented across Midwestern and Southern states including Georgia, where House Bill 481 (HB 481) has now outlawed abortion after embryonic cardiac activity (estimated at 6-weeks since last menstrual period) (1–3). Given this increasingly restrictive abortion environment, the role of full spectrum doulas offering abortion support is paramount but poorly understood.

A full spectrum doula, as defined by the Birthing Advocacy Doula Training (BADT) organization, is “a non-medically trained community care worker who offers support to people during the full spectrum of pregnancy—from preconception, to birth, to abortion, to miscarriage, to adoption, to postpartum” (4). The scope of full spectrum doula care can include supporting clients through in-home visits, hospital or clinic accompaniment, emotional support, pain management, education, and advocacy (4, 5). Birth doula certification is typically a lengthy and expensive process that involves didactic courses as well as practical experience attending births (6). Notably, abortion and full spectrum doula training is largely de-centralized but is provided by doula training agencies like BADT, Dopo, and the Doula Project in New York City (4, 7, 8). Payment for doula services also varies from doula to doula (with individual doulas creating their pricing for each of their services as they see fit), patient to patient, and state to state. In some cases, doulas are funded by insurance (but never for abortion), paid out-of-pocket by their client, or offered for free (pro bono) or through volunteer organizations (4, 9).

The limited existing literature on clinic-based abortion doula care shows there are numerous benefits including improved mental and physical health, respect for reproductive autonomy, and reduced demand on clinicians (11–16). Doulas use similar non-medical services for their abortion clients as they would with other prenatal, birthing or postpartum clients such as “hand-holding, massage, reassurance, providing guidance with breathing, educating about the nature of the procedure or engaging in conversation” [(12), p. 124]. These techniques lead to benefits for clients as well as for physicians and staff providing abortion care. Clients' psychosocial needs are met with abortion doula care and they are “affirmed as moral decision-makers” [(10), p. 111, (11, 13)]. Additionally, in studies looking at doula support during first-trimester surgical abortion, clients reported feeling their educational needs were met, and they expressed gratitude for their doula educating them on abortion and post-abortion care (14, 16). For physicians and staff providing abortion care, the presence of an abortion doula in the clinic allows them to focus on technical aspects of the abortion procedure, while knowing that their patient's emotional needs are being met by their doula (15). Providers at high-volume abortion clinics may see many patients throughout the day, but abortion doulas can focus on their single client's physical, emotional, and educational needs, offering continuous support and guidance.

Less is known about community-based abortion doulas outside of clinical settings, particularly in restrictive abortion contexts, including their scope of work, the benefits of that work, and any facilitators or barriers for providing care. Community-based doulas are those that are not affiliated with a hospital or clinic, but rather focus directly on community members, often their own racial/ethnic group or in a particular geographic area (17). In global settings where abortion has been historically restricted including Argentina and Northern Ireland, community-based abortion doulas have served pivotal roles in accompanying people when seeking and accessing care (18, 19). Further research is needed to understand how community-based doulas can further integrate full spectrum care (including abortion support) in restrictive abortion settings—especially for socially marginalized community members.

The Georgia Doula Study (20, 21) is a community-engaged participatory action research project (22) based at the Center for Reproductive Health Research in the Southeast in Emory University's Rollins School of Public Health. Co-led by a doula-researcher and a non-profit community-based maternal and child health organization, the study team conducted in-depth interviews and surveys with doulas in metro-Atlanta, Georgia to understand: (1) How does the doula community in metro-Atlanta view doula-supported abortion services and abortion generally?; (2) How do abortion doulas describe their services?; and (3) What are the facilitators and barriers to accessing abortion doula support in metro-Atlanta?.

## 2. Materials and methods

This mixed methods, community-engaged study is overseen by a statewide doula stakeholder steering committee with representation from community-based doulas, hospital administrators, clinicians, insurance payers, and policymakers. The doula stakeholder steering committee first identified the need for a Georgia Doula Study that interviews and surveys doulas across the state to support policy advocacy for doula access, integration of doula care into hospital systems, and increased community awareness of doula care. The steering committee provided guidance on study design, assisted with recruitment, helped to interpret results, and aided dissemination to community, clinical, hospital, and policy stakeholders.

### 2.1. Study design and recruitment

This project uses a cross-sectional, observational design and concurrent mixed methods (qualitative and quantitative). Participant recruitment and data collection for the larger study occurred from October 2020 to November 2021. For this sub study, participant recruitment occurred specifically during August to November 2021. Participants were purposively sampled and recruited through emails to the doula stakeholder steering committee, abortion clinics, and local reproductive health and justice organizations in metro-Atlanta. The research team originally surveyed and interviewed seventeen doulas between October 2020 and February 2021. In the fall of 2021, additional measures on family planning and abortion were added to the interview guides, and previous participants were re-contacted. Fourteen previous participants were re-interviewed; and three additional participants were interviewed and surveyed for a total of twenty doulas. Notably, some doulas could not be re-contacted for interview, and two discontinued participation in the study due to the nature of questions regarding family planning and racial discrimination. One participant explained they did not see abortion as within the scope of doula care and were made uncomfortable by the survey questions about abortion, while the second participant (white) was uncomfortable with the questions about racism and doula care. All participants were given an additional $20 for completing the additional survey and interview. If a participant completed the survey, but was not available for an in-depth interview, they were still compensated for their time with a $20 gift card. Thematic saturation was reached after 20 participants, when the academic-community study team collectively agreed that no new data were being collected after multiple iterative cycles of collection and analysis.

### 2.2. Procedures

Potential participants were first screened for eligibility, which included practicing as a doula in Georgia for at least six months and being over the age of 18. While not an original criterion of the study, all participants were community-based doulas. Eligible participants were then consented for participation, given a survey link, and scheduled for an in-depth interview lasting on-average 60 min. If participants did not respond after two follow-up outreach emails from a member of the study team, they were deemed unreachable. Survey data was collected using Qualtrics and no identifying information was collected: all names were replaced with anonymous participant ID numbers. Interviews were connected on Zoom, audio recorded, transcribed verbatim, and de-identified. All study participants interacted with one of two graduate research assistants (AL and AS), who were responsible for participant recruitment, screening, and interviewing.

### 2.3. Instruments

#### 2.3.1. Survey measures

The survey (see Supplementary Appendix A) measured demographic information including gender, race/ethnicity, age, economic status, highest level of education, current employment, sexual orientation, and immigration status. Gender was measured as (check all that apply) female/woman, male/man, transgender, genderqueer, nonbinary, or self-identify. Race/ethnicity was measured as (check all that apply) Black/African American, Hispanic/Latinx, Asian/Pacific Islander, American Indian/Alaskan Native/Native Hawaiian, Biracial/multiracial, White, Other (specify), or prefer not to answer. Age was measured as under 25, 25–25, 36–45, 46–55, Over 55. Economic status was measured as “difficulty affording necessities such as education costs, food, clothing, transportation, housing, and medical care” with response categories: yes, currently; yes, in the recent past (within 3 years); yes, in the past for a limited period of time; yes, historically throughout my life; no; or prefer not to answer. Highest level of education was measured as high school or less, some college, graduated college, graduate degree, clinical professional degree, non-clinical professional degree, or other. Current employment was measured as full-time, part-time, not employed, and not looking, and not employed and looking. Sexual orientation was measured as (check all that apply) lesbian, gay, bisexual, queer, straight/heterosexual, don't know/questioning, self-describe, or prefer not to answer. Immigration status was measured as (check all that apply) my parents and grandparents were born in the U.S.; one or more of my grandparents were born in the U.S.; one or more of my parents was born in the U.S.; I was born in the U.S.; or prefer not to answer. This was further categorized into not an immigrant (self, parents, and grandparents born in the U.S.), first-generation immigrant (born in U.S. but not parents or grandparents), or second-generation immigrant (self and one/more parents born in the U.S. but not grandparents).

Participants were also asked how long they had been a doula (years and months), what trainings and/or certifications they have completed, and what kind of doula services they provide. Doula services provided (check all that apply) were preconception/fertility, prenatal, birth, postpartum, abortion, full spectrum, radical/justice, death/grief/loss/bereavement, prison, or other (specify). Preconception/fertility services include supporting clients, who are trying to become pregnant. Prenatal services include supporting clients through their pregnancy before childbirth. Birth doula services can include the prenatal period but focus on emotionally and physically supporting a client through their childbirth. Postpartum services can be wide-ranging and include emotional support, cooking, and cleaning for a client or offering newborn care during the months following a birth. Abortion doulas support clients through pregnancy termination experiences (12). Full spectrum doulas typically offer more comprehensive services, often including prenatal, birth, postpartum, abortion, and miscarriage support (21, 23). Radical/justice doulas can provide a variety of doula services, but with a specific lens of client empowerment, advocacy, and Reproductive Justice (21, 24). Death/grief/loss/bereavement doulas offer emotional services to clients who are either personally dying or are close to a person who was dying or deceased (25). In some cases, this includes clients who had a miscarriage, stillbirth, or infant loss. Participants were also asked what percentage of their client base received which doula services.

All doulas were asked questions that assessed their abortion stigma (See Supplementary Appendix A for survey items). Abortion stigma was measured using a revised version of the Stigmatizing Attitudes, Beliefs, and Actions Scale (SABAS) with 37 items (26). The survey also measured abortion provider stigma (27, 28) through a revised Abortion Provider Stigma Scale (APSS) with 13 items where abortion doulas were asked to “indicate how often you have felt or experienced the following” and given various prompts (Supplementary Appendix A) (27). Additionally, our revised APSS measured 3 subscales: “*disclosure management”* (6 items), *“discrimination”* (2 items) and “*resistance and resilience*” (5 items). “*Disclosure management”* refers to the process of deciding whether to share or hide one's identity as an abortion worker, which can lead to silencing and isolation; “*discrimination*” refers to judgement, poor treatment, and social exclusion because of one's work in abortion care; and “*resistance and resilience”* refers to positive aspects of abortion work including commitment to a mission to serve those in need (27, 28). Higher scores on the overall APSS and the “*disclosure management”* and “*discrimination*” subscales indicated higher levels of stigma while a high “*resistance and resilience*” subscale score indicated higher resilience (27).

#### 2.3.2. In-depth interview domains

In-depth interviews covering the topics of abortion and contraception were conducted with 20 participants. The domains included abortion doula training, clientele and payment, abortion and contraceptive counseling services, abortion and contraceptive stigma, and experiences of racism or other discrimination (see Supplementary Appendix B). Doulas that reported offering abortion services were asked to go into detail on their abortion and contraception services, while other doulas were asked to describe their thoughts on abortion doula care, abortion, and contraception broadly.

### 2.4. Data analysis

Data analyses on the 20 interviews and survey covering abortion and family planning were conducted from November 2021 to February 2022. Descriptive and bivariate analyses of quantitative data employed Stata v.14 (29). Frequencies and proportions were calculated for categorical variables (ex: type of care provided), while means with standard deviations were calculated for continuous measures (ex: abortion provider stigma). Independent t-tests were conducted to analyze group differences (e.g., abortion attitudes by type of doula).

In-depth interview transcripts were cleaned of errors and de-identified before coding was conducted *via* online, qualitative software Dedoose v.7.0.23 (30). Team members used memo-ing techniques to summarize the main content of each interview and identify the top ten emerging topics of interest from all interviews. The comprehensive list of emerging topics augmented an existing codebook from the previous round of data collection. The team used a semi-deductive coding structure that resulted in both deductive and inductive codes about abortion, contraception, training, doula scope of work, building doula businesses, underserved populations, payment, challenges, client stories, benefits of doula care, medical outcomes, ways to improve doula care, COVID-19, and discrimination. Two members of the study team coded 1/3 of the qualitative transcripts together, met to reach consensus and clarify the codebook and its application, then individually coded the remaining transcripts separately. The coding group then developed analytic memos for each code in order to develop themes within and across codes. This process was supported by additional analyses within Dedoose including code co-occurrence and matrices.

### 2.5. Ethical considerations

The study protocol and materials were reviewed by the Emory Institutional Review Board (IRB) for human subjects' ethical clearance, and the study was deemed exempt because the human subjects' identities are not easily identified, and any disclosures would not place the subjects at risk of damages [see rule 45 CFR 46.104(d)(2i((2ii)].

## 3. Results

Five key themes were identified: doulas of all kinds center reproductive autonomy, abortion doulas play important roles in reproductive autonomy, doulas have mixed feelings about contraceptive counseling services, abortion doulas provide diverse services and carry numerous benefits, and abortion doula experience challenges including stigma but offer solutions.

### 3.1. Demographic information

The doulas sampled for this research project were diverse in terms of race/ethnicity, gender identity, age, and other key demographic information. As shown on Table 1, about half of the doulas were Black/African American (9, 45%) and white (8, 40%) with some doulas reporting their race as Hispanic/Latinx (1, 5%) or Other (1, 10%). While nearly all doulas reported their gender identity as cis-gender female, two doulas reported identifying as nonbinary or genderqueer. Additionally, around half of these sampled doulas reported being between 25 and 35 years of age (8, 40%), never experienced economic difficulty (10, 50%), and had attained a college degree (9, 45%). Nearly all doulas reported being employed full-time (12, 60%), being straight/heterosexual (16, 80%), spoke English as their primary language (18, 90%), and did not identify as an immigrant (17, 85%).

**Table 1:** Baseline Characteristics of Community-based Doulas Surveyed and Interviewed in metro-Atlanta Georgia from October 2020 to February 2022 (*N*=20)

Variable	Frequency	Percent
Race/Ethnicity		
Black or African American	9	45%
White	8	40%
Hispanic or Latinx	1	5%
Multiracial	2	10%
**Gender Identity**		
Cis-gender Female	18	90%
Nonbinary or Genderqueer	2	10%
**Age**		
Under 25	1	5%
25-35	8	40%
36-45	7	35%
46 or Older	4	20%
**Economic Status**		
Prefer not to say	1	5%
Currently experiencing economic difficulty	1	5%
Experienced economic difficulty in the past/historically	3	15%
Experienced economic difficulty temporarily	5	25%
Never experienced economic difficulty	10	50%
**Education**		
Some college/technical degree	4	20%
Non-clinical professional degree	2	10%
Graduated college	9	45%
Clinical professional degree	2	10%
Graduate degree	3	15%
**Employment**		
Yes, full-time or part-time	15	75%
No, not looking for employment	3	15%
No, looking for employment	2	10%
**Sexuality**		
Straight/heterosexual	16	80%
Bisexual	1	5%
Queer	2	10%
Lesbian	1	5%
**Primary Language**		
English	18	90%
Other	2	10%
**Immigration Status**		
Not an immigrant	17	85%
First generation immigrant	3	15%

The participants of this study varied in their doula service characteristics from their time to serving as a doula, the type of services they offer, and whether they were certified (Table 2). About half of the participants (9, 45%) had been serving as a doula for 1–3 years and a quarter (5, 25%) for more than 3 and up to 9 years. A third of the doulas reported offering services for less than 1 year (2, 10%) or more than 9 years (4, 20%). A majority (14, 70%) of doulas reported being certified. When asked about their available doula services, participants ranged widely in the types of services they offered. In fact, doulas often reported that their scope of work included more than one type of care. These services included preconception/fertility (7, 35%), prenatal (9, 45%), birth (17, 85%), postpartum (12, 60%), abortion (7, 35%), full spectrum (8, 40%), radical/justice (4, 20%), and death/grief/loss/bereavement (4, 20%).

**Table 2:** Summary of doula service characteristics of community-based doulas surveyed and interviewed in metro-Atlanta Georgia from October 2020 to February 2022 (*N* = 20).

Variable	Frequency	Percent
**Time as Doula**		
Less than 1 year	2	10%
1–3 years	9	45%
More than 3 and up to 9 years	5	25%
More than 9 years	4	20%
**Type of Doula**		
Preconception/Fertility	7	35%
Prenatal	9	45%
Birth	17	85%
Postpartum	12	60%
Abortion	7	35%
Full Spectrum	8	40%
Radical/Justice	4	20%
Death	4	20%
Certified	14	70%

### 3.2. Doulas of all kinds center reproductive autonomy

All participating doulas were asked questions about abortion and contraceptive counseling services in the communities that they served, which revealed a theme that the doula community supports reproductive autonomy for their clients including (largely) abortion. While most participants were outwardly supportive of the role of abortion doulas, this support did not always translate to an interest in incorporating abortion or contraceptive counseling as part of their regular doula offerings.

#### 3.2.1. The role of an abortion doula in reproductive autonomy

As demonstrated in Table 2, participants varied widely in the characteristics of their doula services, with only 7 self-identifying as abortion doulas and 8 as full spectrum. None of the abortion doulas were based in clinics, but rather worked as private, community-based doulas that found their clients through social media, word-of-mouth, or knowing them personally. Regardless of their scope of work, all participants were asked to describe the role of an abortion doula, including how the larger doula community perceived those who provided abortion services. Most surprising was the overall support of abortion services from non-abortion doulas. One such doula who offered exclusively birth and postpartum services, Brenda, stated

*“I think there should be a doula for everything… I just feel like [for] everything, especially important things around family, doulas are amazing. I’m in awe of abortion doulas. I’m happy to hear that there is such a thing.”*

Despite Brenda's role as primarily a birth/postpartum doula, she voiced support for doulas that provided abortion services. Other non-abortion doulas also seemed supportive of abortion doula care and the potential benefits for clients. One such non-abortion doula, Taylor, commented on the opportunity abortion doulas had to provide all-options counseling to clients for them to make an empowered decision,

*“I think that mothers need to be given all of their options before they make a decision to have an abortion. So, I feel like doulas do need to be there to support if they choose to have an abortion, but on the other hand … let’s find resources of people who can support you if you’re low income. Let’s find an adoption agency if you want to go that route. I don’t think that abortion is the only answer or the only option for a lot of people.”*

This call for all-options counseling continued with non-abortion doulas describing what they thought abortion services looked like and how they personally viewed abortion doulas. Annie, a doula serving primarily birth and postpartum clients, expressed her thoughts on the abortion process and the role of the abortion doula,

*“I think it’s a difficult decision to make [having an abortion], and that they [abortion clients] need support in a lot of ways during that process … holding their hand while they’re making that decision, helping them get the resources and then also being there for them during and then those weeks to months afterwards.”*

#### 3.2.2. Mixed feelings on contraceptive services

While a majority of participants asserted that a doula's role is to support reproductive autonomy, some did not see the doula scope of work as including contraceptive counseling. About half (9, 45%) of the participants reported offering family planning counseling, a third (6, 30%) were trained in contraceptive counseling and some (2, 10%) were not currently offering contraceptive counseling services but were interested in the future (Table 3). Doulas were mixed on when to begin providing family planning services with just under half (8, 40%) simply stating that anytime was the right time to provide family planning support.

**Table 3:** Summary of contraceptive counseling services by community-based doulas surveyed and interviewed in metro-Atlanta Georgia from October 2020 to February 2022 (*N* = 20).

Variable	Frequency	Percent
Offers Contraceptive Counseling	9	45%
Trained in Contraceptive Counseling	6	30%
Does not currently offer contraceptive counseling, but is interested	2	10%
**Contraceptive Counseling Timing**		
Prenatal	5	25%
Anytime	8	40%
Client's Request	4	20%
Postpartum	2	10%
Outside Scope	1	5%

Brenda explained why she doesn't offer contraceptive counseling and hasn't pursued training in contraceptive counseling,

*“It doesn’t feel like it falls under the umbrella of the scope of a doula. Unless it happens to come up, you know, and then, of course, talking to supporting families than that would apply but I’m not sure that specifically contraception feels like it would be under the doula umbrella.”*

Some doulas discussed personal experiences of Black and Brown clients being targeted for family planning and referenced the historical mistrust of the medical system in general due to the history of reproductive coercion for Black/brown communities. As a result, many doulas of color were hesitant to bring these topics up with their clients or receive further training on contraceptive counseling.

At the same time, Bailey (a Black full spectrum doula) explained that she provides contraceptive counseling precisely because of the contraceptive coercion Black clients face,

*“Research shows that Black birthing folks are asked about long-acting reversible contraceptives earlier and more often than white folks. So, I make sure to bring it up with my clients as far as the postpartum piece of their birth preferences like, ‘Okay…these are some things for you to think about postpartum and after you have the baby. One of these is birth control, if they haven’t already asked you, they will. And you need to be aware that they will ask about it and you need to start thinking about what you want.’”*

Overall, participants agreed that the role of a doula is primarily to support clients in making informed, empowered decisions through major life changes including births, the postpartum period, or abortions. While this theme of support for reproductive autonomy was evident, contraceptive counseling continued to be a point of disagreement among participating doulas, with some who were interested in offering or were already offering this support and others who had no interest in providing that support in the future.

Doulas that provided abortion or contraceptive counseling services were asked to describe their interactions with clients. Most of these doulas also provided abortion services. Mira described her thoughts on how these services intersect,

*“It’s not an easy decision to have an abortion by any means and most people are not just casually throwing that out there like, ‘Oh it's going to be my form of birth control'. That's not a thing … It is important to talk about sexual health after those things* [abortions]. *If you've had a miscarriage or a loss, what was that journey of getting pregnant? Was that a conscious decision? Was it an accident? How do we prevent future accidents if that's not what you're looking for?”*

Other abortion doulas reported not offering contraceptive counseling services but were interested in beginning that conversation with their clients. Alex, a postpartum, birth, and abortion doula, talked about opportunities to engage postpartum clients in family planning counseling,

*“I know that that's something postpartum folks have a hard time figuring out, how to prevent pregnancies after just having a pregnancy … some people believe, ‘If I breastfeed, I won't get pregnant’. And it's like, well, you might!”*

### 3.3. Abortion doula services and benefits: *“Holding space”* and much more

All abortion doula participants were asked to describe their services and experiences with clients. While none of these doulas reported supporting client in an abortion clinic, they described a range of services they provide as well as how their services benefited clients overall.

#### 3.3.1. The scope of abortion doula services

Abortion doulas described their services as mainly walking clients through the process of abortion by providing physical comfort, emotional support, educational resources, and *“holding space”* for clients to process their abortion experience—that is, an emotional presence that communicates a felt message of trust, empowerment, witnessing, and understanding (16). Nicole described her usual abortion services as first working with the client

*“… to formulate a plan. So that way they experience a reflecti[on] of whatever they want. Like I said, everybody's abortion is not the same … some of them are at home. Some of them are at the hospital, it just really depends on how it's done and what they need. What level of comfort they need. Sometimes they need the physical comfort and support of being there.”*

Several abortion doula participants affirmed the need for clients to be given space and resources to make the decision that is best for them. Bailey stated,

*“… in a person's life being pregnant or choosing to have an abortion or even going through the fertility process, that's just one small part of their life. They have 20,000 other things going on and the doula is the person that's like, ‘Hey, I have this two-hour block set for us to only focus on your pregnancy’ … I think that's a big piece, holding space to acknowledge the thing that's happening, whatever the thing is.”*

Abortion doula participants recognized that the goals of their services were similar to the goals of birth, postpartum, or death doulas. These goals were described as *“holding space,”* providing support, and encouraging education. Nicole, an abortion doula, described this as,

*“… the same thing as a birth doula … they* [the client] *already have whatever process they're going to have lined up, whether it be a D&C* [Dilation and Curettage]*, whether it be taking the pills … they already have it lined up. I just help them formulate a plan. So that way their experience is honored in the way they see fit … my goal is to provide them with an emotional and physical presence and then just being there, allowing them to process their feelings and making sure they have other resources.”*

Abortion doulas also described varying their services based on the specific needs of the client and the context of the abortion experience. While these services varied widely, Bailey, an abortion doula, talked about the experience of a doula-supported abortion being necessary because

*“… for a lot of people abortion can be really isolating … they're the only one whose body is going through the experience of pregnancy and so, even if there is a partner or a friend or a community member who can hold their hand or be there with them, I think it can still feel really isolating. And I think the average person doesn't always know how to hold space for that or how to say the right thing … I think the holding space can be important.”*

The idea of *“holding space”* for clients continued in other abortion doula's descriptions of their services. Doulas who reported offering both birth and abortion services noted how the range of emotions experienced by clients was not always so cut and dry. This made it even more important to hold space for clients to fully process their emotions, physical pain, and/or other reactions to their major reproductive experience. Alex described this as,

*“Yes, it's about holding space for what folks are experiencing physically because it hurts … but also the range of emotion that can exist. And that's not just sadness. Folks assume typically that when folks come home with a baby, they're either through the sky, elated and happy and then if someone just comes home from having a miscarriage or an abortion that they are distraught … there are these assumptions around what emotions look like and that's not it. So, I really try to make sense of what the emotional status of my client is and help them cultivate a care plan.”*

Abortion doulas all described their services as tailored to the client's abortion experience and personal needs which could include emotional, physical, or educational support. *“Holding space”* was an important aspect of their offered services, allowing for clients to process their abortion experience without judgement or stigma.

#### 3.3.2. Abortion doula benefits and client stories

While abortion doulas described their care as being personalized to their client's needs, several key benefits emerged throughout the discussion of their work. Reagan, an abortion and full-spectrum doula, discussed these benefits as,

*“I believe that it's having that outside person that's not going to have a judgment. That you can share your real, raw emotions with and know that no matter what decision you choose to make, they're still going to be there… to be able to help you find the right clinic… helping find funding because there's a lot of people who don't have the funding to get an abortion… explaining to somebody what's going to happen and the things after.”*

Reagan's overview of abortion doula care benefits is important as it shows that while abortion doulas may seem to only be serving clients in a short, time-sensitive window, the benefits of their care expand to before and after a client's abortion experience. Bailey shared a specific abortion client's story, highlighting the benefits of doula support before, during, and after a procedural abortion,

*“My client was 24 or 25 weeks along and we had to travel to Maryland because I think the cut off in Georgia is 20 weeks or 22 weeks* [at the time]* … we raised the money to pay for the Airbnb and all the things. I helped them fundraise to be able to cover the abortion and lodging and travel and all that, and then I also served as their abortion doula. It was a two-day procedure, so I traveled with them and then we were in the Airbnb together. I went to the clinic with them - I couldn't go in because COVID, but I was basically in the waiting room for the entire two days. They* [the abortion providers] *let me come in during the actual procedure … and hold my client's hand. And then the aftercare as far as making sure that the anesthesia wears off okay and that they have enough food and water and things like that. I made a belly oil … to rub on their belly and cabbage leaves on their breasts where it felt uncomfortable or felt painful and then I followed up a couple of weeks later.”*

Bailey's client's story highlighted the wide range of benefits that abortion doula care offers clients during their abortion experience, especially when facing restrictions that force inter-state travel. From navigating those restrictions, raising funds, and even physically supporting where possible, the benefits are impactful and crucial to ensuring a client has access to a safe and timely abortion. Alex also shared an experience supporting an abortion client and what the benefits of doula care looked like,

*“I think being able to process is really important, like being able to just take time and space to process. And sometimes that looks like saying nothing and literally saying nothing. I don't think that my client and I spoke much at all that night, like with everything that we were doing in the space and we sat together. She got a back massage from me … We did yoga, we painted, we created a fort in the living room and engulfed ourselves in things that were stress-relieving and comforting. I think it's having a container to process and hold emotion.”*

These stories highlight the ways in which abortion doulas are able to effectively meet the needs of their clients and improve their overall abortion experiences.

### 3.4. Abortion doula challenges and solutions

Accessing and providing abortion doula services do not come without challenges. When asked to describe the challenges clients may face in accessing their services, abortion doulas overwhelmingly agreed on three major challenges: affordability of services, lack of awareness, and abortion stigma.

#### 3.4.1. Financial challenges

Doulas explained that one of the main challenges, especially for clients who need to pay out-of-pocket for their abortion, was finances. Due to the inability of clients to afford both an abortion and a doula for that abortion, many abortion doulas in this study provided their services pro bono. Imani, a long-time abortion doula, discussed this in more detail,

*“The major challenge, I would say, is just them* [the client] *being able to pay for it … the client's ability to afford it, to pay for it, and to count it as a necessity … it's tough … like I said, every abortion doula client that I've had was pro bono … and of course I did that so that I can get the experience, but also because the need was there and I don't like to turn people away just because they can't pay.”*

This challenge to afford abortion doula services was even more difficult when considering the cost of an abortion procedure and particular client circumstances. Reagan highlighted the factors contributing to the issue of funding by stating that

*“… a lot of young people don't have, like, an extra $250 to $500 or whatever laying around, especially if they've already had to access care related to the thing. Like if they've needed to go and get an abortion … I think it's definitely financial because I know right now, if I got pregnant, I wouldn't be able to afford a doula. And I am one.”*

Reagan points out that most people, even doulas, would not be able to afford an abortion procedure and doula support. This is a major challenge to accessing abortion doula support, especially for low-income, young, or otherwise impacted clients.

#### 3.4.2. Lack of awareness

Another main challenge was lack of awareness of abortion doula services. Abortion and full-spectrum doula, Mira, described this challenge in the context of the client accessing care,

*“… most people who are getting abortions aren't looking for abortion doulas. That's the big thing … it's not a common practice, it's not something that's easily advertised or sold as a service.”*

Abortion doulas then speculated that providers were also unaware of abortion doulas and their potential benefits. Lisa discussed abortion provider lack of awareness as,

*“I've never spoken with providers who provide abortion services. I'm not sure that it's in their lexicon of what an abortion doula is … when you even say the word ‘abortion doulas' they [abortion providers] would probably say, ‘It's not anything I've ever heard’.”*

Abortion provider lack of awareness can impact the abortion experience of clients, especially when looking for ways to be supported during their abortion experience. Even more challenging is the lack of awareness that doulas and birth workers have regarding the ability to support clients during abortion experiences. Alicia, a doula interested in providing abortion services, described her first time meeting an abortion doula as,

*“I had already been a doula and training and stuff, and I had never met … someone who was an abortion doula and* [they talked] *to me about that* [abortion doula support] *and I was just like, ‘Oh my gosh’ … So yeah. I definitely think not a lot of doulas are offering this type of service.”*

Lack of awareness also led to misconceptions of abortion doula support by non-abortion doulas. Annie, a birth and postpartum doula who expressed interest in learning more about abortion doula support, talked about the volunteering she does related to abortion,

*“I do volunteer at a place… it's technically an anti … I teach classes, I don't do the counseling side of it because they're a pro-life clinic. But they gave me little tidbits and things like that, I'm not trying to convince them* [clients] *not to have an abortion, so we don't necessarily align there.”*

Faith-based doulas, like Taylor, also expressed an interest in abortion doula work but only after the abortion experience under the assumption that abortion patients will struggle with their decision:

*“It* [abortion doula work] *is something that I'm interested in. I, obviously as a Christian, I don't agree with abortion. But I would like to be in a professional place and be able to assist if somebody is struggling after what happened … after they made that decision, something like that.”*

#### 3.4.3. Abortion stigma

Misconceptions around what abortion doulas do like those highlighted in the previous section, stem from larger abortion stigma. Abortion ban policies, such Texas's SB 4 and SB 8 bills and Georgia's embryonic cardiac activity (estimated 6 weeks) abortion ban following the Supreme Court decision to overturn *Roe v. Wade*, perpetuate abortion stigma. Several doulas reflected on the impact of restrictive bans on their doula services. Imani stated,

*“… with all the legislation that's being passed … maybe trying to figure out the workarounds and making sure that we don't get in trouble or sued. How we will be able to help people and not endanger our own selves … I'm really concerned about that. I don't want to – if we* [Georgia] *turn into Texas, I don't want to be sued by some random John walking down the street for $10,000 because of my job.”*

Georgia also has a history of restrictive abortion bans (e.g., mandatory counseling, mandatory waiting period, and at first a 22-week limit now brought down to the estimated 6 weeks) that impact the way that abortion doulas interact with clients and abortion providers. Lisa explained,

*“… there are very few providers that could give any information or even would give any information for fear of retribution or backlash on abortion services, especially here in Georgia.”*

Survey results indicated that stigmatizing attitudes, beliefs, and actions about abortion (SABAS) were low with a mean of 22.29 out of an 18 to 40 scale (Table 4), where higher scores indicate more stigmatizing attitudes, beliefs, and actions about abortion. Abortion doulas reported slightly lower mean SABAS than other kinds of doulas (20.8 vs. 23.1), but these differences were not statistically significant (*t* = .65, *P* = .53)

**Table 4:** Abortion attitudes, beliefs, and actions Among community-based doulas surveyed and interviewed in metro-Atlanta Georgia from October 2020 to February 2022 and their experiences of abortion provider stigma.

Variable	Obs	Mean	Std. Dev.	Min	Max
Stigmatizing Attitudes, Beliefs, and Actions Scale (SABAS) Total	14	22.29	6.27	18	40
Abortion Providers Stigma Scale (APSS) Total	7	24	3.32	17	27
APSS Disclosure Management	7	10.86	3.34	7	15
APSS Resilience	8	7	2.67	4	11
APSS Discrimination	8	5.86	.83	5	7

At the same time, survey results revealed a relatively high overall abortion provider stigma scale (APSS) score with a mean of 24.00 out of a 17 to 27 scale (Table 4). Abortion doulas reported moderate disclosure management (mean score of 10.86 out of a 7 to 15 scale), low to moderate discrimination (5.86 out of a 5 to 7 scale), and moderate resistance and resilience (mean of 7.00 out of a 4 to 11 scale) (Table 4).

When doulas reflected on the possible stigma perpetrated by the larger doula community, most believed that their doula community was accepting of abortion doulas. However, some like Annie felt there were negative perceptions of abortion doulas:

*“Probably not well … because a lot of doulas are, although some doulas are very open, I think a lot of doulas come from like upper middle-class families that are … you know. They just wouldn't do that in our area*.”

This stigma was not just felt from the doula community, but also from the participant's larger community of friends, family, and spiritual leaders. Regan described,

*“I got a phone call from my spiritual teacher, “Oh my gosh, you cannot say that! You cannot say that you’re promoting abortions!”. And I said, I’m not promoting anything! I am saying, if you are in that situation, I am here to help.”*

Despite stigma felt by abortion doulas, participants described their desire to continue working in the abortion space as both a doula and advocate. Alex described this desire in the context of their work as both an abortion and birth/postpartum doula,

*“…* *there are just too many reasons that abortion care should be accessible. You’re not going to change my mind about that. And I think what really confuses people with me in particular is when I go from saying that abortion care should 10,000% be accessible, and I’m like, oh, yes, but natural birth should also be 10,000% accessible* *… I can be pro-abortion and also be pro-birth.”*

In stating that doulas can work both in abortion and birth services, Alex and other abortion doulas once again expressed that the doula's role is to support a person's reproductive health experience even in the face of considerable stigma. When asked about how about how doula work can improve in Georgia, many acknowledged the stigma and obstacles to healthcare that many pregnant people face in the United States. Alex states,

“*There's an active attempt to rid our country of the ability to end a pregnancy or to have an abortion. And that is very scary. I think that abortion care is going to be looking different soon and again, like I started off saying, I think that abortion care and sexual reproductive health care is honestly something that should be community based. It's not something that has to happen in a hospital or medical setting … you don't have to go through all the obstacles of getting health care … for something that could be very vulnerable … they* [clients] *deserve to be respected through that and held with integrity*.”

This need for change is echoed in other doula's responses. While this question is asked with the intention of seeing how doulas can best support their clients, some doulas envisioned doula work to fully empower clients to make the decisions necessary for their sexual and reproductive health. Bailey describes this vision of how doula work can change as,

“…* people want to take control of their own health. I think people know that doulas are important, and doulas are great, and people also want to take back their own health and we need to think about what are the ways we can equip people to do that.*”

## 4. Discussion

The Georgia Doula Study is a unique community-engaged participatory action research project that explores abortion doula care from the perspectives of all types of doulas in a restrictive abortion context. This mixed methods study identified important themes and recommendations for improving practice and policy in the increasingly restrictive U.S. abortion landscape. Lessons learned from this study can be widely applied to restrictive abortion settings globally.

To date, the majority of abortion doula literature discusses clinic-based abortion doulas and their in-clinic services; the current study fills an important gap by focusing on community-based abortion doulas. Previous studies primarily focused on how in-clinic abortion doula support can mitigate pain and emotional discomfort during first trimester procedural abortions (14, 16). Participants in this current study described the abortion doula support they provide primarily outside of clinic settings. This included supporting clients before, during, and after abortion with securing funds, providing information, talking through all of the options, physical support, and post-abortion care at home. Our findings align with and augment recently published media articles and commentaries discussing the impact of abortion doula services, typically in response to issues such as COVID-19 and the proliferation of abortion restrictions in the U.S (31–33). Some argue that community-based abortion doula services can overcome stigma and restrictions by providing factually correct educational materials to their clients and supporting them in finding accessible and safe abortion. Internationally, lay community health workers have already been shown to provide informational support and procurement of pills for self-managed abortion (34). COVID-19 has made it even more necessary for community care workers like doulas to provide support during a potentially isolating and stigmatizing abortion experience, especially in areas where restrictive abortion laws are being implemented (33). Moreover, studies about abortion restriction impacts on marginalized communities—like the reproductive justice community-led Georgia Medication Abortion study with Black and Latinx women—emphasize the importance of abortion doulas in educating and supporting disadvantaged pregnant people to access safe abortion care (35).

Most of the participating doulas endorsed abortion care as one way to support pregnant people's reproductive autonomy through an often-stigmatized reproductive health decision. Non-abortion doulas expressed interest in supporting abortion clients, but were not currently doing so. However, it was unclear if this was due to lack of awareness (about opportunities to get trained and provide care) or stigma surrounding abortion work. In the future, doula training and certifying organizations must include abortion and full spectrum support in the scope of work. Similarly, efforts to coordinate abortion care nationally must include community-based abortion and full spectrum doulas, who are connecting patients to information and care. Given the changing post-*Roe* landscape, abortion and full spectrum doulas are increasingly vital resources for people accessing abortion, especially in restricted and criminalized settings like Georgia.

In the U.S. and globally, restrictive abortion policies embody, exacerbate, and enact abortion stigma, which is felt not just by abortion patients but also providers and—the current findings would suggest—doulas (28, 36, 37). This study is the first to measure stigmatizing abortion attitudes of doulas using the SABAS (26) as well as the abortion provider stigma felt by abortion doulas using the APSS (27). Our results show that, overall, doulas do not have stigmatizing attitudes, beliefs, or actions about abortion which suggests they are supportive of their clients, regardless of pregnancy outcome. On the other hand, the moderate to high abortion provider stigma scores suggest that abortion doulas are experiencing stigma, particularly in the form of disclosure management more so than overt discrimination. This suggests that abortion doulas are having to carefully decide when and how to disclosure or hide their abortion work, despite their resistance and resilience against stigma.

In discussing how to improve full spectrum doula care in Georgia and beyond, many abortion and full spectrum doulas spoke about doula work in general needing to evolve to maximize client autonomy in making reproductive health decisions. Specifically, this could include teaching clients how to “doula themselves” or empowering abortion clients, especially, with the information and skills to be their own advocates. This overarching need to reevaluate the role of doula work in the hypermedicalization of reproduction is critical and has been previously discussed in both the gray and scientific literatures (8, 38). Notably, volunteer abortion doula collectives have been instrumental in supporting the abortion experiences of people throughout the U.S., regardless of age or economic background (39, 40). While study participants did not report being a part of any such collective, many expressed the desire to connect with other full spectrum/abortion doulas, especially as restrictive abortion policies are enacted. As the movement towards full spectrum and abortion doula services continues, it will be important for doula training and certifying organizations, abortion doula collectives, and doula policy advocates to be intentional about how they serve abortion clients.

### 4.1. Limitations and delimitations

The current study has a number of limitations, which must be considered when interpreting the results and implications. First, this is a qualitative study with 20 doulas and therefore is not intended to be generalized to broader populations or to prove causality. Second, the recruitment strategies—primarily through the doula stakeholder steering committee and abortion clinics in metro-Atlanta—might have introduced sampling bias, but there is no standard list of all doulas in the metro area and, therefore, a more systematic recruitment process was not feasible. This study's approach allowed the team to leverage partnerships with community-based organizations and abortion providers. Finally, at least one doula did not participate in the in-depth interviews about abortion and full spectrum doula care—and it is possible more doulas like them never began the study—because of anti-abortion sentiments. It is therefore possible that our results do not reflect the full range of doula perspectives on abortion and full spectrum care but rather the perspectives of doulas who were comfortable answering questions about abortion.

### 4.2. Recommendations and conclusions

The practice implications of this study include (1) increased awareness of and funding for full spectrum doula care, (2) organizational support for existing full spectrum doula collectives across the U.S., (3) engagement of all doulas in full spectrum care through increased opportunities for training in abortion care and contraceptive counseling, and (4) abortion de-stigmatization efforts at the community-level. The doula community and general public need to better understand what full spectrum doula care is and how to access abortion doula support. Additionally, financial support (e.g., from abortion funds, insurance companies, private donors) is needed so clients can pay for abortion doula services or to facilitate abortion doulas offering pro bono services while still being compensated for their skills and labor. Full spectrum doula collectives exist across the U.S., but more funding and organizational support are needed to ensure their sustainability and reach (8, 39). Such collectives could serve as a space to train doulas in abortion and contraceptive services as well as a space to be in community with other doulas that are interested in ensuring reproductive autonomy for their clients, regardless of pregnancy outcome. Doula training and certifying organizations must work to include abortion and contraceptive counseling in their scope of education for all doulas. By supporting all clients through the entire spectrum of reproductive health experiences, doulas can ensure that clients feel adequately supported in their full reproductive autonomy. Finally, abortion de-stigmatization efforts are needed at the community level broadly and within the doula community, specifically. Supportive interventions like the Providers Share Workshop (which has been tested domestically, in Latin America, and in Africa) could also reduce the stigma load on abortion doulas in the U.S. and globally (37).

This study also highlights important lessons and potential opportunities for future research. Primarily, this study demonstrates the value and importance of community-engaged participatory action research, especially in the realm of abortion and contraception care. Second, future studies need to further explore the perspectives of clients who are served by abortion and full spectrum doulas. Understanding how clients view and value abortion doula care would inform how abortion doula support can meet ever-changing needs in the years to come. Third, future studies must explore full spectrum doula care in rural areas and with immigrant communities.

Finally, it is also important to recognize that abortion and full spectrum doula care in the U.S. will continue to rapidly evolve given that federal abortion protections were overturned and state-level abortion bans like Georgia's can now be enacted. In particular, studies from the U.S. and globally have shown that self-managed abortion using medication can be safe and effective given the right amount of support including from doulas and community health workers (34, 41, 42). Abortion doulas can offer pivotal emotional, informational, logistical, and physical support during abortion, even in restricted settings and in this new era.

## Data Availability

The de-identified data supporting the conclusions of this article will be made available by the authors upon request, without undue reservation.
