# Supplementary material for: “I can be pro-abortion and pro-birth”: Opportunities and challenges for full spectrum care among doulas in Georgia
Source: Front Glob Womens Health. 2023 Mar 1;4:966208. doi: 10.3389/fgwh.2023.966208 (PMC10014539; doi:10.3389/fgwh.2023.966208)
Supplement: Supplementary file 1 [file Datasheet1.docx]

# Appendix A. Survey Instrument

## Survey Instrument

Study ID: ____________

*Demographics*

1. With which of the following races/ethnicities do you identify? Check all that apply:

☐ Black or African American (1)

☐ Hispanic or Latinx (2)

☐ Asian or Pacific Islander (3)

☐ American Indian, Alaskan Native, or Native Hawaiian (4)

☐ Biracial or Multiracial (5)

☐ White (6)

☐ Other (specify) (7) ______________________________

☐ Prefer not to answer (8)

1. How old are you in years?

☐ Under 25 (1)

☐ 25-35 (2)

☐ 36-45 (3)

☐ 46-55 (4)

☐ Over 55 (5)

1. Have you had difficulty affording necessities such as education costs, food, clothing, transportation, housing, and medical care? Check all that apply

☐ Yes, currently (1)

☐ Yes, in the recent past (within 3 years) (2)

☐ Yes, in the past for a limited period of time (for example, while I was a student) (3)

☐ Yes, historically throughout my life (4)

☐ No (5)

☐ Prefer not to answer (6)

1. Are you currently employed? Check all that apply

☐ Yes, full-time (1)

☐ Yes, part-time (2)

☐ No, not looking for employment (3)

☐ No, looking for employment (4)

1. What is the highest level of education you have completed?

☐ High School (1)

☐ Some college (2)

☐ Graduated college (3)

☐ Graduate degree (e.g., MPH, PhD) (4)

☐ Clinical professional degree (e.g., RN, LPN, MD, PA) (5)

☐ Non-clinical professional degree (e.g., GED) (6)

☐ Other (specify) (7) __________________

1. With which of the following genders do you identify? Check all that apply:

☐ Female/woman (1)

☐ Male/man (2)

☐ Transgender (3)

☐ Genderqueer (4)

☐ Nonbinary (5)

☐ Self-identify (please specify) (6): ____________________

☐ Prefer not to answer (7)

1. What is your sexual orientation? Check all that apply:

☐ Lesbian (1)

☐ Gay (2)

☐ Bisexual (3)

☐ Queer (4)

☐ Straight or heterosexual (5)

☐ Don’t know/questioning (6)

☐ Prefer to self-describe (7) _________________________

☐ Prefer not to answer (8)

1. What language do you primarily speak at home? Check all that apply:

☐ Arabic (1)

☐ Chinese (Cantonese, Mandarin, others) (2)

☐ English (3)

☐ French or French Creole (4)

☐ German (5)

☐ Hindi (6)

☐ Korean (7)

☐ Spanish (8)

☐ Tagalog (9)

☐ Vietnamese (10)

☐ Other (Specify) (11): _______________

1. What is your immigration generation status? Check all that apply

☐ My parents and grandparents were born in the United States (1)

☐ One or more of my grandparents were born in the United States (2)

☐ One or more of my parents were born in the United States (3)

☐ I was born in the United States (4)

☐ Prefer not to answer (5)

1. In which Georgia county do you reside? ________________

*Pregnancy Information*

1. Have you ever been pregnant?

☐ No (1) → Go to introduction to doula work and training

☐ Yes (2) → Go to 11.1

11.1. How many times have you been pregnant?

☐ 1 (1)

☐ 2 (2)

☐ 3 (3)

☐ 4 (4)

☐ 5 or more (5)

11.2 How many live children do you have? ______________

11.3 For any of the pregnancies you mentioned above, did you have a doula?

☐ No (1) → Go to introduction to doula work and training

☐ Yes (2) → Go to personal experiences with doulas section

*Personal Experience with Doulas*

For these questions, consider the last time you had a doula:

12. What type of doula services did you receive? Check all that apply

☐ Birth doula (1)

☐ Postpartum doula (2)

☐ Prenatal doula (3)

☐ Abortion doula (4)

☐ Full Spectrum doula (5)

☐ Radical/Justice doula (6)

☐ Death/Grief/Loss/Bereavement doula (7)

☐ Prison doula (8)

13. How satisfied were you with those doula services?

☐ Very unsatisfied (1)

☐ Unsatisfied (2)

☐ Neutral (3)

☐ Satisfied (4)

☐ Very satisfied (5)

☐ Mixed feelings (6) (Explain: ______________________________________________)

14. How valuable were their services?

☐ Not valuable at all (1)

☐ Mostly not valuable (2)

☐ Neutral (3)

☐ Somewhat valuable (4)

☐ Very valuable (5)

15. How did the doula affect your anxiety about the pregnancy?

☐ Negatively affected, increased anxiety (1)

☐ No effect (2)

☐ Positively affected, decreased anxiety (3)

16. How did the doula affect your pain during childbirth?

☐ Negatively affected, increased pain (1)

☐ No effect (2)

☐ Positively affected, decreased pain (3)

17.  How did the doula affect your empowerment during the pregnancy?

☐ Negatively affected, decreased empowerment (1)

☐ No effect (2)

☐ Positively affected, increased empowerment (3)

18.  Did you have any negative experiences with your doula?

☐ No (1)

☐ Yes (2): Please explain _____________________________________

19.  Would you want a doula again?

☐ No (1)

☐ Yes (2)

*Introduction to Doula Work and Training*

20. How long have you been a doula? (Check all and type amount)

- _____ Years  (1)
- ________Months (2)

21. What kind of doula do you identify as? Check all that apply

☐ Birth doula (1)

☐ Postpartum doula (2)

☐ Prenatal doula (3)

☐ Abortion doula (4)

☐ Full Spectrum doula (5)

☐ Radical/Justice doula (6)

☐ Death/Grief/Loss/Bereavement doula (7)

☐ Prison doula (8)

☐ Other (Specify): ____________

22. How many clients (of each kind) have you been a doula for? (Check all and type amount)

☐ Birth doula (1) ______

☐ Postpartum doula (2) ______

☐ Prenatal doula (3) _____

☐ Abortion doula (4) _____

☐ Full Spectrum doula (5) _____

☐ Radical/Justice doula (6) ______

☐ Death/Grief/Loss/Bereavement doula (7) _____

☐ Prison doula (8) _____

☐ Other (Specify) (9): ____________

22. What, if any, doula training have you completed?

☐ Doulas of North America (DONA) International (1)

☐ CAPPA Childbirth and Postpartum Professional Association (2)

☐ ALACE – Association of Labor Assistants and Childbirth Educators (3)

☐ BirthWorks International (4)

☐ Childbirth International (5)

☐ HypnoBirthing (6)

☐ N/A (7)

☐ Other (Specify) (8): _________

23.  What, if any, doula certification have you completed?

☐ Doulas of North America (DONA) International (1)

☐ CAPPA Childbirth and Postpartum Professional Association (2)

☐ ALACE – Association of Labor Assistants and Childbirth Educators (3)

☐ BirthWorks International (4)

☐ Childbirth International (5)

☐ HypnoBirthing (6)

☐ N/A (7)

☐ Other (Specify) (8): _________

*Family Planning Attitudes*

24. Please read each of the following statements and indicate how much you disagree or agree:

1. People behave differently toward a teen whom they know has used modern family-planning methods
2. Young women who use modern family planning are promiscuous
3. Teens who use modern family planning are viewed as bad girls
4. Modern family planning is not acceptable for unmarried women
5. Modern family-planning methods have bad effects on a woman’s health

- Agree (1)
- Neutral (0)
- Disagree (0)

25. Please tell me whether or not you think it should be possible for a pregnant woman to obtain a legal abortion if...

a. If there is a strong chance of serious defect in the baby?

b. If she is married and does not want any more children?

c. If the woman’s own health is seriously endangered by the pregnancy?

d. If the family has a very low income and cannot afford any more children?

e. If she became pregnant as a result of rape?

f. If she is not married and does not want to marry the man?

g. The woman wants it for any reason

- Yes (1)
- No (2)
- Don’t Know (3)

26. Do you personally think it is wrong or not wrong for a woman to have an abortion...

a. If there is a strong chance of serious defect in the baby?

b. If she is married and does not want any more children?

c. If the woman’s own health is seriously endangered by the pregnancy?

d. If the family has a very low income and cannot afford any more children?

e. If she became pregnant as a result of rape?

f. If she is not married and does not want to marry the man?

g. The woman wants it for any reason

- Always Wrong (1)
- Sometimes Wrong (2)
- Neutral/Don’t Know (3)
- Wrong Only Sometimes (4)
- Not Wrong At All (5)

27. Please read each of the following statements and indicate how much you disagree or agree:

1. A woman who has an abortion is committing a sin
2. Once a woman has one abortion, she will make it a habit
3. A woman who has had an abortion cannot be trusted
4. A woman who has an abortion brings shame to her family
5. The health of a woman who has an abortion is never as good as it was before the abortion
6. A woman who has had an abortion might encourage other women to get abortions
7. A woman who has an abortion is a bad mother
8. A woman who has an abortion brings shame to her community
9. A woman who has had an abortion should be prohibited from going to religious services
10. I would tease a woman who has had an abortion so that she will be ashamed about her decision
11. I would try to disgrace a woman in my community if I found out she’d had an abortion
12. A man should not marry a woman who has had an abortion because she may not be able to bear children
13. I would stop being friends with someone if I found out that she had an abortion
14. I would point my fingers at a woman who had an abortion so that other people would know what she has done
15. A woman who has an abortion should be treated the same as everyone else.
16. A woman who has an abortion can make other people fall ill or get sick
17. A woman who has an abortion should be isolated from other people in the community for at least 1 month after having an abortion.
18. If a man has sex with a woman who has had an abortion, he will become infected with a disease.

- Strongly Disagree (1)
- Disagree (2)
- Neutral/Don’t Know (3)
- Agree (4)
- Strongly Agree (5)

*Family Planning Doula Services*

28. Do you counsel your clients on family planning?

- Yes (1)
- No (2)

If yes, Explain and give an example (open answer)

________________________________________________________________

If no, would you be interested in counseling your patients?

- Yes (1)
- No (2)

29. Have you received any training in family planning?

- Yes (1)
- No (2)

If yes, describe

________________________________________________________________

30. When do you think is best for doulas to provide family planning counseling? (ex: prenatal, postpartum, immediately after delivery)

________________________________________________________________

Skip Pattern: Continue to Abortion Doula questions if YES to providing abortion doula care, skip to client demographics if non-abortion doula

*Abortion Doula Questions*

Please consider your experiences as someone who works in abortion services. Indicate how often you have felt or experienced the following:

31. People’s reactions to my being an abortion worker make me keep to myself

- All of the time [1]
- Often [2]
- Sometimes [3]
- Rarely [4]
- Never [5]

32. I feel marginalized by other health workers because of my decision to work in abortion care

- All of the time [1]
- Often [2]
- Sometimes [3]
- Rarely [4]
- Never [5]

33. I feel like if I tell people about my work they will only see me as an abortion worker

- All of the time [1]
- Often [2]
- Sometimes [3]
- Rarely [4]
- Never [5]

34. I worry about telling people I work in abortion care

- All of the time [1]
- Often [2]
- Sometimes [3]
- Rarely [4]
- Never [5]

35. It bothers me if people in my community know that I work in abortion care

- All of the time [1]
- Often [2]
- Sometimes [3]
- Rarely [4]
- Never [5]

36. I avoid telling people what abortion care I do for a living

- All of the time [1]
- Often [2]
- Sometimes [3]
- Rarely [4]
- Never [5]

37. I am afraid that if I tell people I work in abortion care I could put myself or my loved ones at risk for violence

- All of the time [1]
- Often [2]
- Sometimes [3]
- Rarely [4]
- Never [5]

38. I am proud that I work in abortion care

- All of the time [1]
- Often [2]
- Sometimes [3]
- Rarely [4]
- Never [5]

39. I feel connected to others who do this abortion care work

- All of the time [1]
- Often [2]
- Sometimes [3]
- Rarely [4]
- Never [5]

40. By providing abortion doula care I am making a positive contribution to society

- All of the time [1]
- Often [2]
- Sometimes [3]
- Rarely [4]
- Never [5]

41. I find it important to share with people that I work in abortion care

- All of the time [1]
- Often [2]
- Sometimes [3]
- Rarely [4]
- Never [5]

42. Newspapers/television take a balanced view about abortion care

- All of the time [1]
- Often [2]
- Sometimes [3]
- Rarely [4]
- Never [5]

43. I feel that patients use me as an emotional punching bag

- All of the time [1]
- Often [2]
- Sometimes [3]
- Rarely [4]
- Never [5]

*Client Demographics*: For the answers to each of these questions please estimate a percent for each demographic group (scroll over for 100% option)

44.  Estimate the racial/ethnic breakdown (in percentage) of your clients (total in column should add up to 100)

|  | 10 | 20 | 30 | 40 | 50 | 60 | 70 | 80 | 90 | 100 |
| --- | --- | --- | --- | --- | --- | --- | --- | --- | --- | --- |
| Black or African American |  |  |  |  |  |  |  |  |  |  |
| Hispanic or Latinx |  |  |  |  |  |  |  |  |  |  |
| Asian or Pacific Islander |  |  |  |  |  |  |  |  |  |  |
| American Indian, Alaskan Native, or Native Hawaiian |  |  |  |  |  |  |  |  |  |  |
| Biracial or Multiracial |  |  |  |  |  |  |  |  |  |  |
| White |  |  |  |  |  |  |  |  |  |  |
| Other |  |  |  |  |  |  |  |  |  |  |

45.  Estimate the age breakdown (in percentage) of your clients (total in column should add up to 100)

|  | 10 | 20 | 30 | 40 | 50 | 60 | 70 | 80 | 90 | 100 |
| --- | --- | --- | --- | --- | --- | --- | --- | --- | --- | --- |
| Under 25 |  |  |  |  |  |  |  |  |  |  |
| 25-35 |  |  |  |  |  |  |  |  |  |  |
| 36-45 |  |  |  |  |  |  |  |  |  |  |
| Over 45 |  |  |  |  |  |  |  |  |  |  |

46.  Estimate the socioeconomic status breakdown (in percentage) of your clients (total in column should add up to 100)

|  | 10 | 20 | 30 | 40 | 50 | 60 | 70 | 80 | 90 | 100 |
| --- | --- | --- | --- | --- | --- | --- | --- | --- | --- | --- |
| Upper |  |  |  |  |  |  |  |  |  |  |
| Upper Middle |  |  |  |  |  |  |  |  |  |  |
| Middle |  |  |  |  |  |  |  |  |  |  |
| Lower Middle |  |  |  |  |  |  |  |  |  |  |
| Lower |  |  |  |  |  |  |  |  |  |  |

47.  Estimate the highest level of education breakdown (in percentage) of your clients (total in column should add up to 100)

|  | 10 | 20 | 30 | 40 | 50 | 60 | 70 | 80 | 90 | 100 |
| --- | --- | --- | --- | --- | --- | --- | --- | --- | --- | --- |
| High School |  |  |  |  |  |  |  |  |  |  |
| Some college |  |  |  |  |  |  |  |  |  |  |
| Graduated college |  |  |  |  |  |  |  |  |  |  |
| Graduate degree (e.g., MPH, PhD) |  |  |  |  |  |  |  |  |  |  |
| Clinical professional degree (e.g., RN, LPN, MD, PA) |  |  |  |  |  |  |  |  |  |  |
| Non-clinical professional degree (e.g., GED) |  |  |  |  |  |  |  |  |  |  |
| Other |  |  |  |  |  |  |  |  |  |  |

48.  Estimate the number of pregnancies breakdown (in percentage) of your clients (total in column should add up to 100)

|  | 10 | 20 | 30 | 40 | 50 | 60 | 70 | 80 | 90 | 100 |
| --- | --- | --- | --- | --- | --- | --- | --- | --- | --- | --- |
| 1 |  |  |  |  |  |  |  |  |  |  |
| 2 |  |  |  |  |  |  |  |  |  |  |
| 3 |  |  |  |  |  |  |  |  |  |  |
| 4 |  |  |  |  |  |  |  |  |  |  |
| 5 |  |  |  |  |  |  |  |  |  |  |
| Over 5 |  |  |  |  |  |  |  |  |  |  |

*Racism/Discrimination Questions*

Here are some situations that can arise at work. Please tell me how often you have experienced them during the LAST 12 MONTHS.

49. How often are you UNFAIRLY given the jobs that no one else wants to do?

- Once a week or more (1)
- A few times a month (2)
- A few times a year (3)
- Less than once a year (4)
- Never (5)

50. At work, when different opinions would be helpful, how often is your opinion not asked for?

- Once a week or more (1)
- A few times a month (2)
- A few times a year (3)
- Less than once a year (4)
- Never (5)

51. How often are you watched more closely than other doulas?

- Once a week or more (1)
- A few times a month (2)
- A few times a year (3)
- Less than once a year (4)
- Never (5)

52. How often does the medical team use racial or ethnic slurs or jokes?

- Once a week or more (1)
- A few times a month (2)
- A few times a year (3)
- Less than once a year (4)
- Never (6)

53. How often do members of the medical team direct racial or ethnic slurs at you?

- Once a week or more (1)
- A few times a month (2)
- A few times a year (3)
- Less than once a year (4)
- Never (5)

54. How often do other doulas use racial slurs or ethnic jokes?

- Once a week or more (1)
- A few times a month (2)
- A few times a year (3)
- Less than once a year (4)
- Never (5)

55. How often do other doulas direct racial or ethnic slurs or jokes at you?

- Once a week or more (1)
- A few times a month (2)
- A few times a year (3)
- Less than once a year (4)
- Never (5)

56. How often do you feel that you have to work twice as hard as others work?

- Once a week or more (1)
- A few times a month (2)
- A few times a year (3)
- Less than once a year (4)
- Never (5)

57. How often do you feel that you are ignored or not taken seriously by the medical team?

- Once a week or more (1)
- A few times a month (2)
- A few times a year (3)
- Less than once a year (4)
- Never (5)

58. How often do others assume that you work in a lower status job than you do and treat you as such?

- Once a week or more (1)
- A few times a month (2)
- A few times a year (3)
- Less than once a year (4)
- Never (5)

59. How often has a doula with less experience and fewer qualifications gotten more clients than you?

- Once a week or more (1)
- A few times a month (2)
- A few times a year (3)
- Less than once a year (4)
- Never (5)

60. How often have you been unfairly humiliated in front of others at work?

- Once a week or more (1)
- A few times a month (2)
- A few times a year (3)
- Less than once a year (4)
- Never (5)

*Clients, Cost, and Other Logistics*

61.  How do you primarily find your doula clients? Check all that apply:

- Fewer than preferred (1)
- Actual number preferred (2)
- More than preferred (3)
- Don’t know (4)

62.  How do you primarily find your doula clients? Check all that apply:

☐ Personal website (1)

☐ Professional doula organization website/registry (2)

☐ Word-of-mouth (3)

☐ Other online forums (4)

☐ Telephone (5)

☐ Health care providers and institutions (6)

☐ Community-based programs (7)

63.  What type of doula practice are you a part of? (check all that apply)

☐ Solo practice (1)

☐ Group practice with 2-4 doulas (2)

☐ Group practice with 5+ doulas (3)

☐ Hospital-based practice (4)

☐ Clinic-based practice (5)

64. How often do you charge clients for your doula services?

- Always (1)
- Sometimes (2)
- Never (3)

65.  How much do you currently charge ($USD) per client for (check all and type amount):

☐ Birth doula (1) ______

☐ Postpartum doula (2) ______

☐ Prenatal doula (3) _____

☐ Abortion doula (4) _____

☐ Full Spectrum doula (5) _____

☐ Radical/Justice doula (6) ______

☐ Death/Grief/Loss/Bereavement doula (7) _____

☐ Prison doula (8) _____

☐ Other (Specify) (9): ____________

66. Do you plan on charging for your doula services in the future?

- Always (1)
- Sometimes (2)
- Never (3)

67.  How much do you think you should be paid (ideally, in order to reach standard of living) per client for (check all and type amount):

☐ Birth doula (1) ______

☐ Postpartum doula (2) ______

☐ Prenatal doula (3) _____

☐ Abortion doula (4) _____

☐ Full Spectrum doula (5) _____

☐ Radical/Justice doula (6) ______

☐ Death/Grief/Loss/Bereavement doula (7) _____

☐ Prison doula (8) _____

☐ Other (Specify) (9): ____________

*Doula Services During COVID*

68.  In what ways have you and your work been affected by COVID-19? (Check all that apply)

☐ Stopped taking on clients (1)

☐ Unable to accompany clients in the delivery room (2)

☐ Limited prenatal and postpartum visits (3)

☐ Increase in client home births (4)

☐ Use of protective equipment (i.e. masks, gloves) when working with clients (5)

☐ My work has not changed as a result of COVID-19 (6)

69.  Are you interested in providing doula services virtually (i.e. video and phone calls)?

☐ Yes (1)

☐ No (2)

70.  Have you provided virtual doula services?

☐ Yes (1)

☐ No (2)

71. Have you provided doula services virtually during the COVID-19 pandemic?

☐ Yes (1)

☐ No (2)

Skip Pattern: Continue to Virtual Doula questions if YES to providing virtual doula care, skip to Beliefs about Doula Services if NO

*Virtual Doula Services During the Pandemic*

72. How many clients have you served virtually since the onset of the pandemic? _______

73. Do any of your clients have difficulties accessing the internet?

☐ Yes (1)

☐ No (2)

☐ Unknown (3)

74. How do you connect with your clients virtually? (check all that apply)

☐ Video calls (Zoom, Microsoft Teams, Facetime) (1)

☐ Phone (2)

☐ Other (3): Specify _________

75. (If Video Calls is selected) What platform do you use for video calls?

☐ Zoom

☐ Skype

☐ Teams

☐ Other: Specify _________

*Beliefs about Doula Services*

76.  For each of the following, mark the answer that you most closely agree with.

|  | Strongly Disagree | Disagree | Neutral | Agree | Strongly Agree |
| --- | --- | --- | --- | --- | --- |
| I believe current pricing of doula services helps me to provide doula services. |  |  |  |  |  |
| I believe current pricing of doula services helps my clients access doula services. |  |  |  |  |  |
| I believe current insurance coverage of doula services helps me to provide doula services. |  |  |  |  |  |
| I believe current insurance coverage of doula services helps my clients access doula services. |  |  |  |  |  |
| I believe current doula training helps me to provide doula services. |  |  |  |  |  |
| I believe current doula training helps my clients access doula services. |  |  |  |  |  |
| I believe current doula certification requirements help me to provide doula services. |  |  |  |  |  |
| I believe current doula certification requirements help my clients access doula services |  |  |  |  |  |
| I face challenges in starting my doula business. |  |  |  |  |  |
| I face challenges in building my client base for my doula business. |  |  |  |  |  |
| I face challenges in making enough profit to continue my doula business. |  |  |  |  |  |

*Possible Changes for Doula Service Reimbursement*

77.  How interested would you be in Medicaid reimbursement for your doula services?

☐ Not interested at all

☐ Mostly uninterested

☐ Neutral

☐ Somewhat interested

☐ Very interested

☐ Mixed feelings (Explain: ______________________________________________)

78.  How interested would you be in Georgia doulas being classified as Community Health Workers who are reimbursed through Department of Public Health?

☐ Not interested at all

☐ Mostly uninterested

☐ Neutral

☐ Somewhat interested

☐ Very interested

☐ Mixed feelings (Explain: ______________________________________________)

# Appendix B. In-Depth Interview (IDI) Guide

## New Participant IDI Guide (Abortion Doula)

*I. Opening Questions*

1. Why did you become a doula?

*II. Training*

1. On the survey you mentioned you received X,Y,Z training. Tell me more about your training experience.
   1. Probe:
      1. If abortion training is listed:
         1. Where did you complete abortion doula training?
         2. What topics were covered in your training?
      2. If abortion training is not listed:
         1. Without specific training in abortion doula care, how have you developed the skills needed to support abortion clients?
   2. Additional Probes
      1. How did you pay for doula training you received? (ex: out-of-pocket, funded by an organization, grant-funded, etc.)
      2. What training have you received around working with diverse populations including Black, Latinx, non-English speaking, refugee, and LGBTQ clients?
      3. What additional training would you like to receive?

*III. Practice and Clientele*

1. On the survey you mentioned you provided X,Y,Z doula services (prenatal/birth, post-partum, abortion). What do those services typically involve?
   1. Probe: Can you walk me through your typical services with X clients?
   2. Probe: Can you walk me through your typical services with Y clients?
   3. Probe: Can you walk me through your typical services with Z clients?
2. How did you build your doula practice?
   1. Probe:
      1. How did you develop your client base? (social media, word of mouth, website, established doula practices/programs, physician or midwife referrals)
      2. What relationships (maternal health organizations, physician practices, community doula organizations) helped you start your practice?
3. Where do you provide services?
   1. Probe: Are they affiliated with a clinic, hospital, or community-based organization? Does she go to where the client is receiving care? etc.
4. Are there any demographic groups you wished you could be a doula for but have not been able to reach?
   1. Follow-up: What do you think are the challenges for these groups in accessing doula services?
   2. Follow up: What could make it easier for these groups to access doula services?

*IV. Payment*

1. Non-volunteer doulas: On the survey you said you do not work as a volunteer doula. In general, how do you work with clients to ensure they can afford doula services—if at all?
   1. Probe:
      1. Do you use a sliding scale?
      2. Do you make referrals to other doulas?
2. Volunteer doulas: On the survey you said you are a volunteer doula. Why did you decide to volunteer your doula services as opposed to charge for them?
   1. Probe: What motivates you to be a volunteer doula?

*V. Doula Roles in Family Planning*

1. What needs do your clients have related to contraception?
   1. Probe: Do they need more information, can’t afford it, don’t know where to get it?
2. What, if any, training have you received in contraceptive counseling?
   1. Probe: What kind of (additional or new) family planning training would you need or want?
3. What, if any, counseling do you provide your clients about contraception and birth control?
   1. Probe: If none, would you be interested in providing family planning counseling in the future?

*VI. Abortion Care Counseling Questions for abortion doulas*

1. Why did you become an abortion doula?
   1. Probe: What kind of additional, or new abortion training would you like to receive?
2. Can you describe in more detail the services you provide as an abortion doula?
3. Where do you provide abortion services?
4. What are the benefits of having an abortion doula?
5. How do you feel you are perceived from the larger prenatal, birth, and postpartum doula community?
6. How do you think abortion care providers perceive you as an abortion doula?
7. What challenges do you face in providing abortion doula care?
   1. General probes:
      1. What are some challenges to building a sustainable doula business?
      2. How is your dynamic with your client’s medical care providers?
      3. Do you feel there are adequate supports from your doula community? (mentorship opportunities, networking, etc.)
   2. Probe based on survey answers:
      1. On your survey you mentioned that you feel marginalized by other health workers because of your decision to work in abortion care. Can you tell me more about that?
      2. On your survey, you mentioned that it bothers you if people in your community know that you work in abortion care. Can you tell me more about that?
      3. On your survey, you mentioned that you feel abortion clients use you as an emotional punching bag. Can you tell me more about that?

*VII. Racism and Discrimination Qs for all doulas*

1. Can you tell me a time you witnessed or experienced discrimination as a doula?
   1. Probe based on their survey answers:
      1. you’ve been watched more closely than others in your work as a doula
      2. you’ve been humiliated during your work as a doula
      3. you’ve heard racial slurs or ethnic jokes in your work
   2. General probes:
      1. Racial discrimination (of yourself or the client)
      2. Discrimination because you’re a doula
      3. Age discrimination (of yourself or the client)
2. How does your race influence the interactions you have with the medical team?
   1. Probe: Can you provide examples of when you felt your race was a factor in how the medical team treated you?
3. How does the race of your client influence your experience with the medical team?
4. How does your race influence the interactions you have with your clients?
   1. Probe: For clients of your race?
   2. Probe: For clients of a different race?
5. What training, if any, did you receive about providing culturally competent care?
   1. Probe:
      1. For example, care specific to the needs of a racial/ethnic group?
      2. What additional training would you like to receive?

*Client Stories*

1. Tell me a story about a time when you had a lot of impact on a client, or when a client had a lot of impact on you.
   1. Probe: For example, a life-changing story?
   2. Probe: For example, a story you can’t forget?
   3. Probe: For example, a story highlighting the value of doulas?
2. Describe a time when your doula services impacted maternal and infant health.
   1. Probe: emotional wellbeing around pregnancy and delivery
   2. Probe: empowerment during pregnancy and delivery
   3. Probe: birth outcomes and complications

*Concluding Questions*

1. How can we improve doula services in Georgia?
   1. Probes:
      1. Awareness of doula services and their benefits
      2. Reimbursement through insurance
      3. Training
      4. Mentorship
      5. Integration into clinical services, improving dynamics with L&D staff (Note: some do not want it clinically integrated and prefer home births)
2. In an ideal world, what would doula work look like in Georgia?
   1. Probe:
      1. community health worker models
      2. insurance reimbursement
      3. hospital doulas
      4. community-based doulas
      5. partnerships at the state-level and local-level

Do you know any doula in Georgia that provide abortion services and would be interested in participating in this study?

## New Participant IDI Guide (Non-Abortion Doula)

*Opening Questions*

1. Why did you become a doula?

*II. Training*

1. On the survey you mentioned you received X,Y,Z training. Tell me more about your training experience.
   1. Probe:
      1. How did you pay for training?
      2. What training have you received around working with diverse populations including Black, Latinx, non-English speaking, refugee, and LGBTQ clients?
      3. What additional training would you like to receive?

*III. Practice and Clientele*

1. On the survey you mentioned you provided X,Y, and Z doula services (prenatal/birth, post-partum, etc.). What do those services typically involve?
   1. Probe: Can you walk me through your typical services with X clients?
   2. Probe: Can you walk me through your typical services with Y clients?
   3. Probe: Can you walk me through your typical services with Z clients?
2. How did you build your doula practice?
   1. Probe:
      1. How did you develop your client base? (social media, word of mouth, website, established doula practices/programs, physician or midwife referrals)
      2. What relationships (maternal health organizations, physician practices, community doula organizations) helped you start your practice?
3. Where do you provide services?
   1. Probe: Are you affiliated with a clinic, hospital, or community-based organization? Do you go to where the client is receiving care? etc.
4. Are there any demographic groups you wished you could be a doula for but have not been able to reach?
   1. Follow-up: What do you think are the challenges for these groups in accessing doula services?
   2. Follow up: What could make it easier for these groups to access doula services?

*IV. Payment*

1. Non-volunteer doulas: On the survey you said you do not work as a volunteer doula. In general, how do you work with clients to ensure they can afford doula services—if at all?
   1. Probe:
      1. Do you use a sliding scale?
      2. Do you make referrals to other doulas?
2. Volunteer doulas: On the survey you said you are a volunteer doula. Why did you decide to volunteer your doula services as opposed to charge for them?
   1. Probe: What motivates you to be a volunteer doula?

*V. Doula Roles in Family Planning*

1. What needs do your clients have related to contraception?
   1. Probe: Do they need more information, can’t afford it, don’t know where to get it?
2. What, if any, training have you received in contraceptive counseling?
   1. Probe: What kind of (additional or new) family planning training would you need or want?
3. What, if any, counseling do you provide your clients about contraception and birth control?
   1. Probe: If none, would you be interested in providing family planning counseling in the future?

*VI. Abortion Care Counseling Questions for non-abortion doulas*

1. What needs do your clients have related to abortion care counseling?
2. What, if any, training have you received in abortion care counseling?
   1. Probe: What kind of additional or new abortion care training would you need or want?
3. What, if any, counseling do you provide to your clients regarding abortion services?
   1. Probe: If none, would you be interested in providing abortion services counseling in the future?
4. There are some doulas that provide abortion care services to their clients. How do you feel about these abortion doulas?
   1. Probe: How does the larger doula community perceive abortion doulas?
5. Prenatal/birth doulas: What happens if your birth/prenatal doula client experiences complications during a pregnancy?
   1. Probe:
      1. A miscarriage
      2. Decides to terminate their pregnancy
      3. Stillborn or dies shortly after birth

*VIII. Challenges*

1. What is the most challenging part of your job as a doula?
   1. Probes
      1. What are some challenges to building a sustainable doula business?
      2. How is your dynamic with your client’s medical care providers?
      3. Do you feel there are adequate supports from your doula community? (mentorship opportunities, networking, etc.)

*IX. Racism and Discrimination Questions*

1. Can you tell me a time you witnessed or experienced discrimination as a doula?
   1. Probe based on their survey answers:
      1. you’ve been watched more closely than others in your work as a doula
      2. you’ve been humiliated during your work as a doula
      3. you’ve heard racial slurs or ethnic jokes in your work
   2. General probes:
      1. Racial discrimination (of yourself or the client)
      2. Discrimination because you’re a doula
      3. Age discrimination (of yourself or the client)
2. How does your race influence the interactions you have with the medical team?
   1. Probe: Can you provide examples of when you felt your race was a factor in how the medical team treated you?
3. How does the race of your client influence your experience with the medical team?
4. How does your race influence the interactions you have with your clients?
   1. Probe: For clients of your race?
   2. Probe: For clients of a different race?
5. What training, if any, did you receive about providing culturally competent care?
   1. Probe:
      1. For example, care specific to the needs of a racial/ethnic group?
      2. What additional training would you like to receive?

*Client Stories*

1. Tell me a story about a time when you had a lot of impact on a client, or when a client had a lot of impact on you.
   1. Probe: For example, a life-changing story?
   2. Probe: For example, a story you can’t forget?
   3. Probe: For example, a story highlighting the value of doulas?
2. Describe a time when your doula services impacted maternal and infant health.
   1. Probe: emotional wellbeing around pregnancy and delivery
   2. Probe: empowerment during pregnancy and delivery
   3. Probe: birth outcomes and complications

*Concluding Questions*

1. How can we improve doula services in Georgia?
   1. Probes:
      1. Awareness of doula services and their benefits
      2. Reimbursement through insurance
      3. Training
      4. Mentorship
      5. Integration into clinical services, improving dynamics with L&D staff (Note: some do not want it clinically integrated and prefer home births)
2. In an ideal world, what would doula work look like in Georgia?
   1. Probe:
      1. community health worker models
      2. insurance reimbursement
      3. hospital doulas
      4. community-based doulas
      5. partnerships at the state-level and local-level
